# Confidence—More a Personality or Ability Trait? It Depends on How It Is Measured: A Comparison of Young and Older Adults

**DOI:** 10.3389/fpsyg.2016.00518

**Published:** 2016-04-18

**Authors:** Karina M. Burns, Nicholas R. Burns, Lynn Ward

**Affiliations:** School of Psychology, University of AdelaideAdelaide, SA, Australia

**Keywords:** confidence, self-confidence, metacognition, calibration, older-adults

## Abstract

The current study (*N* = 244) compared two independently developed and substantively different measures of self-confidence; a self-report measure, and a measure described as “online.” Online measures are confidence-accuracy judgments made following each item on a cognitive task; in the current study, online measures were yoked to tasks of fluid and crystallized intelligence. The self-report and online measures had not previously been compared, and it was unknown if they captured the same self-confidence construct. These measures were also compared to self-efficacy and personality for the purpose of defining self-confidence as an independent construct, as well as to clarify the primary comparison. This study also aimed to replicate previous findings of a stable factor of confidence derived from online measures. An age comparison was made between a young adult sample (30 years and under) and an older adult sample (65 years and over) to determine how confidence functions across the lifespan. The primary finding was that self-report and online measures of confidence define two different but modestly correlated factors. Moreover, the self-report measures sit closer to personality, and the online measures sit closer to ability. While online measures of confidence were distinct from self-efficacy and personality, self-report measures were very closely related to the personality trait Emotional Stability. A general confidence factor—derived from online measures—was identified, and importantly was found in not just young adults but also in older adults. In terms of the age comparison, older adults had higher self-report self-confidence, and tended to be more overconfident in their judgments for online measures; however this overconfidence was more striking in the online measures attached to fluid ability than to crystallized ability.

## Introduction

Confidence has recently been deemed important because of its predictive validity for academic achievement (Stankov et al., 2013). The finding that non-cognitive factors, specifically confidence, predict academic achievement, is a hopeful one because when compared to IQ, the self-confidence trait is potentially malleable and, therefore, could become an important target of intervention to improve academic achievement (Stankov et al., 2012).

There are two methodologies that measure self-confidence, a self-report measure, and a measure described as “online.” Online self-confidence has been so-called because of the metacognitive function, which informs these types of judgments. The online measure is a post-task question, which asks the respondent to rate how confident they are that their answer was correct. These two types of measures have developed independently and while both describe self-confidence, there are clear differences in both their measurement and their application. Stankov's (1999) early conceptualization of self-confidence saw it as fitting somewhere between an ability trait and a personality trait. It may be that the position of self-confidence on the spectrum between ability and personality depends on the way in which it is measured.

That is, it may be that when measured via self-report, self-confidence sits closer to personality traits, but when measured online it sits closer to ability traits (cf. emotional intelligence, e.g., Burns et al., 2007). Recent research (Stankov and Lee, 2008) which considered confidence in relation to abilities, personality and metacognition found some evidence to support this argument, finding online self-confidence to be more closely related to cognitive abilities than personality traits. The authors suggested that different types of confidence may exist: a cognitive confidence versus a social confidence that can be measured as a part of personality. The following will detail the nature of both the self-report and online measures, including their theoretical backgrounds and supporting research.

The self-report self-confidence measure is administered in questionnaire format and the respondent is asked to report their levels of confidence in both specific (e.g., social, academic) and general domains. Self-report confidence measures capture an overarching self-assessment of confidence, requiring reflection upon personal experiences and tendencies. Measures of this nature have primarily been developed and validated in student populations and used for the assessment of confidence in domains pertaining to academic achievement (Schohn and Shrauger, 1995; Sander and Sanders, 2003; Pulford and Sohal, 2006; Betz and Borgen, 2010). Typically, items on self-report self-confidence scales ask respondents to consider their confidence in particular tasks or areas and select the extent to which they agree with statements or questions in relation to their own behavior.

The two self-report measures used here are the Personal Evaluation Inventory (PEI; Schohn and Shrauger, 1995) and the Trait-Robustness of Self-Confidence Inventory (TROSCI; Beattie et al., 2011). These measures were chosen because they were the most succinct, had good psychometric properties, and were the most relevant and adaptable to the populations of interest. Although, other self-report measures of confidence are available (see Stankov et al., 2015), they were not deemed suitable for use with an older adult population because they focus on confidence in an academic context only.

The PEI was developed for use in a college sample; its 54 items reflect domains rated as important to this particular population, for example, academic, social and romantic. This scale is made up of six domain specific subscales as well as a mood subscale and a general self-confidence subscale, which measures self-confidence generally across time and tasks. In the development of the PEI, both specific and general subscales of the PEI were compared to personality as measured by the NEO PI (Costa and McCrae, 1985). There were statistically significant correlations between the general self-confidence subscale and both Extraversion (0.38) and Neuroticism (−0.60); there were, as well, significant negative correlations with the Neuroticism facets of Depression (−0.62) and Anxiety (−0.57). A factor analysis indicated that the general subscale was equivalent to the six domain specific subscales, in that the general subscale did not add to the explained variance of the self-confidence construct (Schohn and Shrauger, 1995). Due to this equivalence, and for the purpose of conciseness, here we use only the general subscale. One finding on the PEI which is particularly relevant to the current study is highlighted by Stankov et al. (2015, p. 163) in their evaluation of self-confidence measures: “Significant and positive subscale intercorrelations were indicative of the existence of a general confidence factor.”

The TROSCI was developed to measure the robustness of self-confidence in the face of disconfirming experiences; that is, it is a measure of confidence stability. This eight-item measure focuses on how confidence is affected by a poor result, and how much confidence fluctuates on a day-to-day basis. This measure was developed for use specifically with athletes. Three studies developed and validated this measure within college athlete samples across a range of sports (Beattie et al., 2011). It was found that athletes who scored highly on this measure had higher confidence stability, and were more resilient in the face of disconfirming experiences. Importantly, while targeted at athletes, a large majority of the items in this measure are general in content and therefore easily adaptable to suit a general population.

The measurement of self-confidence online developed from early research in decision making which employed the use of accuracy ratings in relation to items on cognitive tests (Stankov et al., 2013). These measures have been described as “online” because they relate to a just completed task, and involve a metacognitive judgment of accuracy. Online confidence ratings can either be discreet estimates, or confidence interval estimates. Discreet estimates can be expressed as either verbal categories along a Likert scale (e.g., “very unsure” to “very sure”), or as percentage ratings along a scale (e.g., 0–100%). The most common online measures come in the form of a numerical confidence rating yoked to individual items of an ability task. Following each item the respondent is asked to give a confidence accuracy rating in response to the question “How confident are you your answer is correct?”

When using online confidence measures an individual's calibration score is calculated as the difference between confidence ratings averaged across a task and actual accuracy (i.e., percentage of items answered correctly), and they indicate how well confidence judgments map on to task performance and provide insight into cognitive biases. Using online confidence measures, it has been established that there is a general confidence factor (see Kleitman and Stankov, 2007) which “reflects the habitual way in which people assess the accuracy of their cognitive performance” (Stankov et al., 2015, p. 186). Factor analyses utilize the confidence score when examining the dimensionality of the self-confidence trait because it has been found that while the calibration score is useful for examining group differences it is not a reliable score to use in the context of determining factor structure. Given there is a stable confidence trait it follows that calibration scores will be relatively stable regardless of what the test is measuring: an individual's tendency to be under- or over-confident will carry over to different tasks or domains, regardless of personal skill or experience.

Morony et al. (2013) used an online measure of self-confidence in a study of several self-belief measures including self-efficacy, self-concept and anxiety. An important finding was that, of all the self-belief measures, confidence was most closely related to accuracy. This is explained as being due to the post-task context of the self-confidence measure (Morony et al., 2013); this is in contrast to the predictive nature of the self-efficacy measure. Stankov et al. (2013) confirmed this finding, calling confidence “the best (known) non-cognitive predictor of achievement on cognitive tests” (p. 24) with correlations between confidence and accuracy typically falling between 0.4 and 0.6. Other studies provide support for Stankov et al.'s conclusion (see Kleitman and Moscrop, 2010; Kleitman and Costa, 2014).

The relationship between confidence and academic achievement was further examined by Stankov et al. (2013) using a sample of Singaporean students (*N* = 598, mean age 15.4 years). Regression analysis showed confidence (uniquely and conjointly) to account for 47% of variance on a mathematics achievement test, with a further 11% accounted for by a cognitive ability score taken from the secondary school entrance exam. Moreover, confidence captured most of the predictive variance across all the self-belief measures. Finally, it was noted that while confidence certainly falls within the non-cognitive self-belief domain, it may also have a cognitive element due to its comparatively small loading on the mathematics self-belief factor as compared to other self-belief measures.

There is reason to compare young adults with older adults for the measure of online self-confidence to determine how confidence behaves across the lifespan. There is also no literature available on an age group comparison for the self-report confidence measures discussed above. The self-report measures currently available have been used on student samples; therefore the basis of the age comparison on these measures here is largely exploratory. Based on the most recent findings of Stankov et al. (2015) the trait of self-confidence—derived from an online measure of confidence—is stable across knowledge domains; therefore, individuals should have consistent self-confidence calibration in tests of, for example, both fluid and crystallized intelligence. There has been substantial documentation that while crystallized intelligence remains stable or even increases across the lifespan, fluid intelligence decreases with age (see Horn and Noll, 1994). For this purpose, the online measures of confidence in this study will be attached to tests of both fluid and crystallized intelligence.

Crawford and Stankov (1996) considered confidence judgments on tasks of fluid and crystallized intelligence, short term memory, and perceptual discrimination across the lifespan in a sample aged from 18 to 85 years (*N* = 97). This study found, as anticipated, that older adults performed more poorly on tasks of fluid intelligence, and better on tasks of crystallized intelligence, as compared to young adults. In terms of confidence judgments older adults showed a greater tendency toward overconfidence than young adults “[t]his trend was constant across the fluid intelligence, crystallized intelligence and visual discrimination tasks, despite the differing nature of the changes with age in actual performances on these tasks” (Crawford and Stankov, 1996, p. 99). In spite of the importance of these findings to the current study, it is necessary to treat them as only tentative foundations to current hypotheses; in terms of the expansive literature published by Stankov and colleagues on confidence judgments, this preliminary study encompassed only primitive methodologies, with a small and unequal sample (i.e., older adults were not adequately represented, especially above the age of 75). Moreover, these findings have not since been followed up and require replication with larger samples.

More recently in a cross-sectional study (*N* = 150), Kavé and Halamish (2015) found that in tests of vocabulary—a measure of crystallized ability—older adults out-performed young adults and middle aged adults, and were also significantly better calibrated in both global and item-by-item confidence judgments. The authors suggested that in the older adult sample “confidence judgment is determined not only by participants' perception of their lifelong mastery of vocabulary but also by their experience while performing the task at hand” (p. 5). That is, older adults make use of their metacognitive insights while doing the problem, as well as their previous knowledge about their performance on such tasks, leading to more concordance between accuracy and confidence for this group. These two studies tell a slightly different story in terms of self-confidence calibration across the lifespan. While Crawford and Stankov (1996) found an overconfidence bias in older adults for tasks of both crystallized and fluid intelligence, Kavé and Halamish (2015) suggest that in tasks of crystallized intelligence, older adults are better calibrated than young adults.

The current study compares self-report measures of self-confidence to online measures of self-confidence. These two self-confidence measures have not yet been directly compared to determine if they capture the same construct. Factor analysis will be used to determine the dimensionality of these measures. Either both types of self-confidence measure (self-report and online) will load on the same factor, suggesting that the underlying construct they measure is the same or, alternatively, these measures will fit a two factor model, with self-report and online measures loading on different factors.

Some research has suggested that confidence sits between personality and ability traits (Stankov, 1999); we hope to clarify this description. It may be the case that self-report measures of confidence are indicative of a trait of self-confidence similar in nature to personality, while online measures are more similar to abilities due to the metacognitive component of the confidence judgment. This study seeks to examine the relationship of both measures of self-confidence (self-report and online) with measures of self-efficacy and personality. These comparisons are for the purpose of identifying self-confidence as an independent construct. Additionally there may be further clarification for the primary aim; if online and self-report measures load on two different factors it likely that self-report self-confidence will have stronger correlations with personality traits and online confidence will have stronger correlations with ability traits.

There has been evidence that online measures yield a stable confidence factor, which generalizes across tasks. This study aims to replicate this finding using measures of both fluid and crystallized intelligence in the measurement of online confidence. Some research has suggested a general confidence factor arises from not just online measures, but also self-report measures (Stankov et al., 2015). This possibility will be considered here.

The final aim of this study is to compare young adults with older adults on measures of online confidence. The premise for this comparison is that previous studies have found in some tasks, particularly vocabulary related, older adults are better calibrated in their confidence judgments than young adults (Kavé and Halamish, 2015). This is counter to the earlier finding of Crawford and Stankov (1996) that older adults are more overconfident than young adults in tasks of both fluid and crystallized intelligence. The inclusion of measures of both crystallized and fluid intelligence yoked to online confidence judgments mirrors the study design of Crawford and Stankov (1996). Our interest in these measures arises from findings that the rate of decline in these abilities is markedly different: while crystallized intelligence is stable across the lifespan, fluid intelligence shows decline as a function of age. We are interested to see if older adults maintain good calibration for tasks of fluid intelligence, indicative of metacognitive awareness of cognitive decline, or, if like their performance on these tasks, their calibration will be poorer compared to young adults.

Our first hypothesis is that both self-report and online self-confidence measures are correlated with measures of self-efficacy and personality but these correlations are small-to-moderate; self-confidence is an independent construct.

Our second hypothesis has two parts: (i) self-report measures of confidence are more strongly related to personality than are online measures of confidence: measuring something via self-report will make it more like a personality trait; and (ii) online measures of confidence are more strongly related to ability than are self-report measures of confidence: measuring confidence as related to abilities measures will make it more like an ability trait.

Our third hypothesis is that there is a “general” confidence trait for online measures of self-confidence across tasks of both fluid and crystallized intelligence.

Our final hypothesis has two parts: (i) the older adult sample has comparable or higher accuracy on measures of crystallized intelligence and better calibration than the young adult sample; and (ii) the older adult sample has lower accuracy on measures of fluid intelligence and an increase in miscalibration; specifically, older adults are more overconfident on measures of fluid intelligence than the young adult sample.

## Methods

### Participants

Participants were recruited for a young adult sample (aged 30 years and under) and an older adult sample (aged 65 years and over). Young adults were Level I Psychology students at the University of Adelaide (*n* = 148) who participated for course credit, and a convenience sample (*n* = 33) recruited largely through social networks. After excluding participants who did not meet the age criterion there were a total of *N* = 153 young adults, (*M* = 20.2, *SD* = 2.78 years, range = 16–29). Older adults were recruited from a community sample (*N* = 104), previously recruited to take part in other studies within the School of Psychology. After removing some participants for whom there were substantial missing data there were *N* = 91 older adults (*M* = 73.0, *SD* = 6.04 years, range = 65–100). Participants were required to have a high proficiency in English, and were asked as part of the study whether English was their first language.

## Materials

### Personality

A measure of the five factor model of personality (FFM) was used: The Openness Conscientiousness Extraversion Agreeableness Neuroticism Index Condensed (OCEANIC; Schulze and Roberts, 2006). This measure contains 45 items and asks participants to rate, on a 6-point Likert scale, the frequency with which they engage in each of the behaviors described in the items, with a response of (1) indicating that they never engage in the specified behaviors and (6) indicating that they always engage in the specified behaviors. The reliability of the OCEANIC measure is good (see Schulze and Roberts, 2006) with Cronbach's α for the five factors ranging from 0.77 (Openness) to 0.91 (Conscientiousness and Neuroticism).

### Self-efficacy

Self-efficacy has been defined as a person's belief in their ability to achieve an outcome. To measure self-efficacy, the Generalized Self-Efficacy Scale (GSES; Schwarzer and Jerusalem, 1995) was used. This is a ten-item measure scored on a 4-point Likert scale from (1) not at all true to (4) exactly true. Each item asks about the respondent's perception of their ability to overcome a problem or achieve a goal. Reliability for this measure is good, with Cronbach's α in samples from 23 nations ranging from 0.76 to 0.90 (Schwarzer and Jerusalem, 1995).

### Self-report self-confidence and confidence stability

A two-part questionnaire measure of self-confidence was adapted from the Personal Evaluation Inventory (PEI; Schohn and Shrauger, 1995) and the Trait-Robustness of Self-Confidence Inventory (TROSCI; Beattie et al., 2011). The first part measures general self-confidence and is taken from the six item self-confidence subscale of the PEI. These items are scored on a 4-point Likert scale from (1) strongly disagree to (4) strongly agree. The reliability for the general subscale of the PEI is good, Chronbach's α = 0.71 (Schohn and Shrauger, 1995, *N* = 211). The second part measures confidence stability and is made up of eight items taken from the TROSCI, a scale originally developed to measure self-confidence stability in athletes, but which we have adapted to suit a general adult population. These items are scored on a 9-point Likert scale from (1) strongly disagree to (9) strongly agree. The reliability of the TROSCI is good, Chronbach's α = 0.88 (Beattie et al., 2011, *N* = 268). The reason for using the TROSCI as well as the PEI is that the TROSCI measures the ability to maintain confidence, which captures self-confidence stability rather than general self-confidence, which is measured by the PEI.

### Online self-confidence

Three measures of ability yoked to confidence rating scales were used, two of fluid intelligence and one of crystallized intelligence. The reason for using two measures of fluid intelligence but only one measure of crystallized intelligence was that the two fluid measures were only 12 items each, while the crystallized measure was made up of 34 items. The first fluid intelligence measure is a short form of Ravens Advanced Progressive Matrices (APM; Raven et al., 1998) and comprises 12 items validated by Bors and Stokes (1998) for use as a brief form. This measure has good reliability, Cronbach's α = 0.71. The second measure is the Comprehensive Abilities Battery-Induction (CAB-I), a test of inductive reasoning (Hakstian and Cattell, 1975); the CAB-I is a 12-item measure that asks participants to identify patterns across sets of letters. This measure has good reliability, Cronbach's α = 0.75 (Hakstian and Cattell, 1975). The third measure is of crystallized intelligence (Word Meanings (WM); Raven et al., 1998). WM is made up of 34 items that ask participants to select, from six options, the word closest in meaning to a target word presented. For all three tests, each item is followed by a discreet categorical numerical scale (i.e., an 11 point confidence rating scale from 0 to 100%) where participants are asked to indicate how confident they are that they got the question correct. Technically, confidence scales should range from 100/k to 100 (where k is the number of response choices, e.g., APM has 8 response options, CAB-I has 5, WM has 6). This format would begin the scale at the point corresponding to the probability of a correct answer when simply guessing; however, to avoid confusion a standard 0-100% scale was used and responses were adjusted so that any less than 100/k were set at 100/k. From these three measures and their attached confidence ratings the following were determined: percentage of items correct, average confidence ratings and a calibration score which is calculated as the difference between average confidence and percentage of items correct. Positive calibration scores indicate overconfidence. In the case of incomplete data sets for these tasks, both confidence and calibration scores have been calculated based on percentage correct of attempted items rather than total items. Reliability for online self-confidence ratings is good with Cronbach's α ranging from 0.75 to 0.90 (e.g., Stankov and Crawford, 1996a,b; Jonsson and Allwood, 2003; Kleitman and Stankov, 2007). Jonsson and Allwood (2003) reported test-retest coefficients for calibration scores collected at three time points, separated by 2 weeks, which correlated 0.53 (T1 & T2),0.59 (T2 & T3), and 0.53 (T1 & T3), respectively. Stankov and Crawford (1996a,b) reported Parallel Forms and Odd/Even reliabilities for the calibration scores; the lowest reliability (corrected by the Spearman-Brown formula) was 0.70. Stankov et al. (2015) suggest that the calibration score is not ideal for use in correlational study designs because of its low reliability, but lends itself to between group comparisons, such as age differences in calibration.

### Procedure

There were three versions of the online data collection instrument available varying only in the demographic details collected. The first survey was available to first year Psychology students through the School of Psychology research participation system, the second survey was sent via email invitation to a pool of 200 older adults previously recruited for other studies in the School of Psychology, the third survey was posted via social media, calling for participants aged 30 years and under. The tests were administered to all participants in the following order: OCEANIC, GSES, PEI, TROSCI, APM, CAB.

### Ethical considerations

This study received ethics approval from the University of Adelaide, School of Psychology Human Research Ethics Subcommittee and participants affirmed informed consent prior to commencing their participation.

### Data analysis

Statistical analyses were conducted using R (R Core Team, 2015) and MPlus v7.3 (Muthén and Muthén, 2014).

## Results

Table 1 gives an overview of participant characteristics in both the young and older adult samples. The highest level of education variable had five levels from “did not complete secondary school” to “postgraduate qualification.” Data for this variable are only relevant in the older adult sample; the majority of the young adult sample were Level I Psychology students and of the remainder recruited through social networks (*n* = 33), the majority were currently studying or had obtained a post-secondary qualification. In the older adult sample, level of education is diverse; however 85% of the sample had completed Year 12 and 27% of the sample had obtained a Postgraduate qualification. English was the first language in 87% of the young adult sample and 95% of the older adult sample. The distribution of self-rated health status (measured on a five point scale from “very poor” to “very good”) was similar in both groups, with the largest proportion responding “good” or “very good.” An additional self-report question on dementia, stroke, or head trauma was asked of the older adult sample. An analysis comparing the older adults who had self-reported a diagnosis of dementia, stroke or head trauma (*n* = 7), with the rest of the older adult sample (*n* = 84), found no significant differences and therefore no participants were excluded.

**Table 1:** **Characteristics of participants (*N* = 244) including age, gender, highest level of education, self-reported health status, and first language**.

	***N* (%)^a^**			
	**Young adults (*****n*** = **153)**		**Older adults (*****n*** = **91)**	
Age, Mean (*SD*)	20.2	(2.78)	73.0	(6.04)
Female sex	105	(69)	31	(34)
**Highest level of education**^b^				
Did not complete secondary school	–		14	(15)
Completed year 12	–		13	(14)
Certificate/Diploma	–		30	(33)
Bachelor degree	–		9	(10)
Postgraduate qualification	–		25	(27)
**Self-rated Health Status**				
Very good	59	(39)	28	(31)
Good	72	(47)	51	(56)
Fair	19	(12)	11	(12)
Poor	3	(2)	1	(1)
Very Poor	0	(0)	0	(0)
**First language**				
English	133	(87)	86	(95)
Other	20	(13)	5	(5)

a Values are expressed as total n (%), except age which is expressed as Mean(SD);

b *The majority of young adults were Level I Psychology students. Those who were not (n = 33) were recruited via personal and social networks and the majority were studying or had a post-secondary qualification*.

Table 2 presents descriptive statistics for all the study variables. There were statistically significant differences between the young and older adults on the OCEANIC measure of personality; older adults were higher in Conscientiousness, while younger adults were higher in Extraversion, Agreeableness, and Neuroticism; there was no statistically significant difference in Openness for this sample. These findings are largely consistent with the literature on age differences in personality traits; for example, McCrae et al. (1999, 2000) found age to be correlated positively with Conscientiousness and Agreeableness, and negatively with Extraversion, Openness and Neuroticism and Srivastava et al. (2003) reported older adults to be higher in Conscientiousness and Agreeableness, and lower in Neuroticism and Openness. Consistent with the literature on age differences for measures of crystallized and fluid intelligence, older adults performed significantly better than young adults on the measure of crystallized intelligence (WM) and significantly poorer on one of the measures of fluid intelligence (APM). Interestingly, the second measure of fluid intelligence (CAB-I) behaved differently across the age groups than was expected: there was no significant difference between the groups on percentage correct, confidence rating, or calibration score. Finally, in terms of the calibration scores and consistent with the findings of Crawford and Stankov (1996), older adults were more overconfident in both the WM and the APM. However, the calibration score for APM was substantially higher (i.e., more overconfident) than for WM: 22.9 and 5.07, respectively, (where a score of 0 is perfect calibration). That is, older adults are relatively well calibrated on the WM task, just less so than the young adults, who had a calibration score of 1.06.

**Table 2:** **Descriptive statistics for all dependent variables with comparison between group scores for young (*n* = 153) and older adults (*n* = 91)**.

	**Young adults** ***M*** **(*****SD*****)**		**α**	**Older adults** ***M*** **(*****SD*****)**		**α**	***t*** **(df)**^**a**^		***p***	**Cohen's d**
GSES	30.5	(3.89)	0.85	30.7	(4.12)	0.91	0.28	(180.7)	0.78	0.04
**OCEANIC**										
Openness	32.4	(6.62)	0.78	33.3	(7.09)	0.81	0.97	(179.1)	0.33	0.13
Conscientiousness	37.2	(7.82)	0.88	39.5	(7.04)	0.89	2.38	(205.1)	0.02	0.31
Extraversion	33.3	(7.63)	0.87	31.4	(7.22)	0.84	1.96	(197.6)	0.05	0.26
Agreeableness	43.8	(5.83)	0.87	41.9	(5.39)	0.85	2.58	(201.3)	0.01	0.34
Neuroticism	29.6	(8.03)	0.88	20.9	(4.91)	0.82	10.6	(241.8)	<0.001	1.32
PEI	14.1	(3.50)	0.81	17.1	(3.03)	0.74	6.89	(210.6)	<0.001	0.89
TROSCI	32.1	(13.8)	0.92	44.9	(12.3)	0.86	7.51	(206.9)	<0.001	0.99
**WM**										
Percentage correct	59.0	(15.3)	0.83	76.9	(13.6)	0.85	9.49	(206.4)	<0.001	1.24
Confidence	60.1	(13.9)	0.95	82.0	(12.6)	0.97	12.7	(205.0)	<0.001	1.65
Calibration	1.06	(11.2)	–	5.07	(8.60)	–	3.15	(226.2)	0.002	0.40
**APM**										
Percentage correct	56.6	(23.7)	0.78	39.7	(22.6)	0.69	5.53	(196.7)	<0.001	0.73
Confidence	62.3	(18.6)	0.91	62.7	(2.11)	0.92	0.13	(177.5)	0.90	0.02
Calibration	5.74	(17.7)	–	22.9	(22.5)	–	6.23	(155.5)	<0.001	0.85
**CAB-I**^b^										
Percentage correct	72.0	(26.9)	0.86	75.4	(24.2)	0.83	0.97	(166.6)	0.33	0.13
Confidence	71.9	(22.8)	0.94	76.3	(24.0)	0.96	1.31	(145.4)	0.19	0.19
Calibration	–0.10	(17.6)	–	0.84	(15.2)	–	0.41	(172.1)	0.68	0.06

GSES is the General Self-efficacy Scale, OCEANIC is the Openness, Conscientiousness, Extraversion, Agreeableness, Neuroticism Inventory Condensed, PEI is the Personal Evaluation Inventory, TROSCI is the Trait Robustness of Self-confidence Inventory, CAB-I is the Comprehensive Abilities Battery-Induction;

a Welch's robust t-test, which does not assume equal variances in both groups, was used which results in non-integer degrees of freedom. The same pooling of variance was used in calculation of Cohen's d using R package lsr (Navarro, 2015);

b *The n for the CAB-I is lower than the other measures in the older adult sample (n = 76) because it was the last measure and there was some drop-out in this group*.

Supplementary Material Tables 1, 2 show the correlation matrices for all variables in both the combined sample (Table 1) and for the young and older adult samples separately (Table 2).

### Factor analysis

The main aim concerns the dimensionality of the five self-confidence measures (two self-report and three online). For the combined data from the young and older adults for the five self-confidence measures (PEI, TROSCI, and average confidence ratings from WM, APM, and CAB-I), there are two eigen values greater than one, and both the scree test (Cattell, 1966) and a parallel roots analysis (Horn, 1965) suggest a two factor solution. Repeating the analyses on the young and older adult samples separately also results in a decision favoring a two-factor solution (it is acknowledged that the sample size for the older adult sample is marginal for factor analysis).

To maximize the available sample size for factor analysis a technique that allows the inclusion of covariates within an EFA was used (Exploratory Structural Equation Modelling, ESEM; Asparouhov and Muthén, 2009). Thus, the three self-confidence measures that correlated significantly with age (PEI, TROSCI and WM, see Supplementary Material, Table 1) were regressed on age as part of the estimation of a two-factor EFA solution for the full sample of *N* = 244. The solution was estimated via maximum likelihood with geomin rotation in MPlus v7.3 (Muthén and Muthén, 2014). The factor loadings, factor correlations and regression coefficients are shown in Table 3. The fit of this model was good [χ${}_{\left(3\right)}^{2}$ = 1.46, *p* = 0.69]. It can be seen that the self-report confidence measures and the online measures clearly define two separate factors, which are correlated at *r* = 0.28 (*p* = 0.003). This analysis supports a conclusion that self-report and online confidence measures are largely unrelated to each other.

**Table 3:** **Results of two-factor exploratory structural equation model for five self-confidence measures (*N* = 244)**.

	**Self-report Factor**		**Online Factor**		***h*^2^**
	**Loading**	***p***	**Loading**	***p***	
PEI	0.51	<0.001	0.08	0.280	0.44
TROSCI	0.98	<0.001	0.00	0.320	0.93
WM Confidence	0.06	0.270	0.39	<0.001	0.54
APM Confidence	0.01	0.540	0.72	<0.001	0.53
CAB-I Confidence	−0.17	0.030	0.78	<0.001	0.57
	Regression coefficient	*P*			
PEI on Age	0.39	<0.001			
TROSCI on Age	0.44	<0.001			
WM Confidence on Age	0.61	<0.001			

*The correlation between the Self-report factor and the Online factor is 0.28 (p = 0.003). Abbreviations are as for Table 2*.

### Hypotheses

Hypothesis 1 was that both types of measures of self-confidence are correlated with measures of personality and self-efficacy but these correlations are small-to-moderate; self-confidence is an independent construct. Findings relevant to this hypothesis can be found in Table 4. This table shows correlations along with their 95% confidence intervals for both self-report and online confidence with self-efficacy (as measured by the GSES) and personality (as measured by the OCEANIC).

**Table 4:** **Correlations and 95% confidence intervals for self-efficacy (GSES) and personality (OCEAN) with both self-report and online measures of self-confidence for young (*n* = 144) and older adults (*n* = 91)**.

	**Self-report Self-confidence**				**Online Self-confidence**					
	**PEI**		**TROSCI**		**WM**		**APM**		**CAB-I**	
**YOUNG ADULTS**										
GSES	0.58	[0.46, 0.68]	0.46	[0.33, 0.58]	0.23	[0.08, 0.38]	0.09	[−0.07, 0.25]	0.12	[−0.04, 0.27]
O	−0.03	[−0.19, 0.13]	−0.04	[−0.20, 0.12]	0.12	[−0.4, 0.27]	0.08	[−0.08, 0.24]	−0.04	[−0.20, 0.12]
C	0.26	[0.11, 0.41]	0.15	[−0.01, 0.30]	0.13	[−0.3, 0.28]	−0.03	[−0.19, 0.13]	0.02	[−0.15, 0.18]
E	0.33	[0.18, 0.47]	0.19	[0.03, 0.34]	0.05	[−0.11, 0.20]	−0.09	[−0.24, 0.07]	−0.02	[−0.18, 0.15]
A	0.03	[−0.13, 0.19]	0.00	[−0.16, 0.16]	0.09	[−0.07, 0.24]	−0.04	[−0.20, 0.12]	0.08	[−0.09, 0.24]
N	−0.69	[−0.76, −0.60]	−0.72	[−0.79, −0.63]	−0.22	[−0.37, −0.07]	−0.28	[−0.42, −0.12]	−0.27	[−0.42, −0.11]
**OLDER ADULTS**										
GSES	0.45	[0.27, 0.60]	0.35	[0.16, 0.52]	0.24	[0.04, 0.42]	0.37	[0.18 , 0.53]	0.10	[−0.12 , 0.32]
O	0.12	[−0.09, 0.32]	0.10	[−0.11, 0.3]	0.30	[01, 0.48]	0.15	[−0.06, 0.34]	0.09	[−0.14, 0.31
C	0.20	[−0.01, 0.39]	0.17	[−0.04, 0.36]	0.27	[0.07, 0.45]	0.25	[0.05, 0.44]	0.26	[0.03, 0.46]
E	0.50	[0.32, 0.64]	0.49	[0.32, 0.63]	0.34	[0.14, 0.51]	0.35	[0.16, 0.52]	−0.07	[−0.29, 0.16]
A	0.28	[0.08, 0.46]	0.26	[0.06, 0.44]	0.21	[0.00, 0.40]	0.19	[−0.02, 0.38]	0.16	[−0.07, 0.37]
N	−0.49	[−0.63,−0.32]	−0.47	[−0.62, −0.29]	−0.03	[−0.23, 0.18]	−0.02	[−0.23, 0.18]	0.27	[0.05, 0.47]

*Abbreviations are as for Table 2*.

In both young and older adults the self-report self-confidence measures (PEI and TROSCI) tend to be positively correlated with most measures of personality. The trait of Neuroticism however, has strong negative correlations with both self-report measures, especially in the young adult sample (PEI, *r* = −0.69, TROSCI, *r* = −0.72, both *p* < 0.001). This kind of negative relationship suggests that the self-report measures define something very similar to Emotional Stability (the opposite of Neuroticism). There are some age differences in the relationship between self-report confidence measures and personality traits. For example, in young adults the personality traits Openness and Agreeableness have negligible and non-significant relationships with both the PEI and the TROSCI, ranging from *r* = −0.04 (*p* = 0.63) to 0.03 (*p* = 0.73); however, in older adults, these traits have small positive correlations with self-report self-confidence, ranging from *r* = 0.10 (*p* = 0.29) to *r* = 0.28 (*p* < 0.001). Extraversion has moderate-to-high correlations with self-report measures of confidence in both age groups; this correlation appears to be stronger in the older adult sample. The GSES has moderate-to-high correlations with both self-report confidence measures in both age groups; however, these correlations appear to be stronger in young adults.

Correlations between online measures of self-confidence (WM, APM, and CAB-I) with the GSES and the OCEANIC on the whole are close to zero. This trend is more evident in the young adult sample, where the only notable relationships are negative correlations between Neuroticism and the three online confidence measures, and a small positive relationship between WM and GSES. In the older adult sample, however, there are small-to-moderate correlations across the three online measures with the GSES and the OCEANIC, the highest of these being correlations of Extraversion *r* = 0.34 (*p* < 0.001) and *r* = 0.35 (*p* < 0.001) with WM and APM, respectively. The GSES similarly has correlations of *r* = 0.24 (*p* = 0.02) and *r* = 0.37 (*p* < 0.001) with WM and APM, respectively.

The expectation of Hypothesis 1 that both measures of confidence would have small to moderate correlations with the GSES and the OCEANIC is largely supported. It is evident from these analyses that self-report measures of confidence are more strongly correlated with personality and self-efficacy. This finding will be explored further in Hypothesis 2, which deals with the strength of the relationship between personality and the self-report and online measures of confidence. The expectation of Hypothesis 1 that both measures of confidence would be distinct constructs from personality is not fully supported. While it is clear that the online measures of confidence are not strongly related to self-efficacy or personality, this is not true for self-report measures of confidence. The strong negative correlations of both the PEI and the TROSCI with the trait of Neuroticism suggest that these measures may in fact describe the personality trait of Emotional Stability.

Part (i) of Hypothesis 2 was that self-report measures of confidence are more strongly related to personality than are online measures and part (ii) was that online measures of confidence are more strongly related to ability than are self-report measures of confidence. Therefore, all confidence and ability measures were included in a single EFA in an attempt to clarify the relationships between self-report and online confidence, personality, and cognitive abilities. The criteria for determining the number of factors to retain varied between two and four factors and therefore three ESEM solutions were examined with 2-, 3-, and 4-factors, respectively. In each, the whole sample was used and variables that correlated significantly with age (PEI, TROSCI, Neuroticism, WM Confidence, WM Correct, and APM Correct, see Supplementary Material) were regressed on age as part of the estimation of the solution. The 4-factor solution could not be estimated and was therefore not considered further. The 2-factor solution was estimated but was not satisfactory because four of the five personality traits (O, C, E, and A) were not captured well by either factor. The 3-factor solution was more satisfactory and interpretable and is presented in Table 5.

**Table 5:** **Results of three-factor exploratory structural equation model for five self-confidence measures, five personality measures, and three ability measures (*N* = 244)**.

	**Factor 1**		**Factor 2**		**Factor 3**		***h*^2^**
	**Loading**	***p***	**Loading**	***p***	**Loading**	***p***	
PEI	0.72	<0.001	0.06	0.350	0.00	0.890	0.67
TROSCI	0.71	<0.001	0.00	0.980	0.01	0.890	0.70
O	−0.07	0.430	0.39	<0.001	0.03	0.700	0.15
C	0.16	0.050	0.44	<0.001	0.06	0.350	0.25
E	0.35	<0.001	0.52	<0.001	−0.15	0.040	0.45
A	−0.01	0.690	0.82	<0.001	0.01	0.680	0.67
N	−0.67	<0.001	0.02	0.750	−0.05	0.270	0.75
WM Correct	−0.04	0.560	0.21	0.020	0.26	<0.001	0.37
APM Correct	0.13	0.040	0.13	0.130	0.27	<0.001	0.42
CAB-I Correct	0.05	0.440	−0.10	0.110	0.52	<0.001	0.75
WM Confidence	0.22	<0.001	0.00	0.970	0.54	<0.001	0.50
APM Confidence	−0.15	<0.001	0.00	0.910	0.87	<0.001	0.37
CAB-I Confidence	0.00	0.940	0.05	0.360	0.89	<0.001	0.80
	Regression coefficient	*P*					
PEI on Age	0.40	<0.001					
TROSCI on Age	0.44	<0.001					
N on Age	−0.54	<0.001					
WM Confidence on Age	0.62	<0.001					
WM Correct on Age	0.51	<0.001					
APM Correct on Age	−0.38	<0.001					

*The correlation of F1 with F2 is 0.13 (p = 0.20), of F1 with F3 is 0.11 (p = 0.20), and of F2 with F3 is 0.05 (p = 0.56). Abbreviations are as for Table 2*.

Factor 1 has loadings from PEI, TROSCI, Extraversion, and Neuroticism, these were all statistically significant (*p* < 0.001). Factor 2 has loadings from Openness, Conscientiousness, Extraversion, and Agreeableness, these were all statistically significant (*p* < 0.001). Factor 3 has loadings from WM Correct, APM Correct, CAB-I Correct, WM Confidence, APM Confidence, and CAB-I Confidence, these were all statistically significant (*p* < 0.001). This factor structure is indicative of two separate factors for confidence, one defined by self-report measures of confidence which are related to the personality traits of Extraversion and emotional stability (negative loading on Neuroticism), the other defined by online confidence measures which are related to ability measures.

Hypothesis 3 was that there is a “general” confidence trait for online measures of self-confidence across tasks of both fluid and crystallized intelligence. The EFA solution in Table 5 showed that the ability and confidence measures loaded together on a single factor. Consistent with Kleitman and Stankov (2007), who found that abilities test scores and confidence measures derived from the same abilities tests defined separate factors in their analyses, here, confirmatory factor analysis models (CFA) are fitted which define two factors, one for the abilities measures and one for the confidence measures. Good fit for these models would provide support for Hypothesis 3. CFA models were estimated in MPlus v7.3 (Muthén and Muthén, 2014). First, as for the ESEM analyses, the whole sample was used and variables that correlated significantly with age (WM Confidence, WM Correct and APM Correct, see Supplementary Material) were regressed on age. Second, data for only the young adults were used to estimate the model because Kleitman and Stankov's (2007) analyses were on young adults. Fit statistics for the whole sample were: [χ${}_{\left(9\right)}^{2}$ = 27.2, *p* = 0.001], RMSEA = 0.06, and CFI = 0.98. Fit statistics for the young adult sample were: [χ${}_{\left(5\right)}^{2}$ = 7.92, *p* = 0.16], RMSEA = 0.09, and CFI = 0.99. According to criteria described by Kline (2011, pp. 191–210), the fit of the model in the whole sample is acceptable and in the young adult sample is excellent. These models also fit better than either a model that specifies all variables loading on a single factor (in the whole sample AIC = 11775 vs. 11843; and in the young adults sample AIC = 7491.6 vs. 7549.6, where smaller AIC indicates better fit when comparing non-nested models); or a model with two factors representing fluid versus crystallized measures with both the ability score(s) and the confidence measure(s) loading on their respective factors (in the whole sample the model could not be estimated; and in the young adults sample AIC = 7491.6 vs. 7549.7). These analyses support Hypothesis 3.

Part (i) of Hypothesis 4 was that the older adult sample has comparable or higher accuracy on measures of crystallized intelligence (as measured by WM) and better calibration than the younger adult sample. Findings relevant to this hypothesis can be found in Table 2. The older adults out-performed young adults and this difference was statistically significant (*p* < 0.001). However, counter to expectations, calibration scores showed older adults to be more overconfident than young adults and again the difference was statistically significant (*p* = 0.002). However, while young adults are better calibrated for this task, the score for the older adults lies within the range of plus-or-minus 10, which is considered good calibration (Stankov et al., 2015, p. 183). Part (i) of Hypothesis 4 is partially supported.

Part (ii) of Hypothesis 4 was that the older adult sample has lower accuracy on measures of fluid intelligence (as measured by the APM and the CAB-I), and an increase in calibration discrepancy; specifically, older adults are more overconfident on measures of fluid intelligence than the younger adult sample. Findings relevant to this hypothesis can be found in Table 2. For the first task of fluid intelligence—the APM—young adults out-performed older adults and this difference was statistically significant (*p* < 0.001). There was a substantial difference in calibration scores between the two groups and this difference was also statistically significant (*p* < 0.001); the score for older adults is indicative of a large overconfidence bias. For the second task of fluid intelligence—the CAB-I—older adults out-performed young adults but the difference was not statistically significant (*p* = 0.33). This finding was unexpected and will be discussed in greater detail in the following section. Calibration scores were close to zero for both groups and the difference was not statistically significant (*p* = 0.68). The results from the APM but not CAB-I support Part (ii) of Hypothesis 4.

## Discussion

### Overview

The current study had the primary aim of considering the relationship between two conceptually different measures of self-confidence. The self-report and online measures of confidence developed independently and have not been directly compared before. This study used two different self-report confidence measures (PEI and TROSCI), and three different cognitive ability tasks, one of crystallized intelligence (WM) and two of fluid intelligence (APM and CAB-I) yoked to online confidence judgements. A secondary aim of the study was to compare both self-report and online confidence measures to self-efficacy and personality, this aim is two-fold, firstly to define confidence as an independent construct from these measures, and secondly for the clarification of the main aim; it was anticipated that self-report confidence would be more related to personality and online confidence would be more related to ability. Previous research has suggested a stable confidence factor derived from online confidence measures, this study aimed to replicate this finding. Finally, an age comparison was made between a young adult sample (aged 30 years and under) and an older adult sample (65 years and over). This comparison was for the purpose of examining how confidence functions across the lifespan, particularly in regards to online confidence which was attached to both crystallized and fluid ability measures, known to behave differently across the age trajectory (i.e., fluid ability declines with age, while crystallized ability remains stable).

### Key findings

A key finding of this study was that self-report and online measures of confidence define two separate factors. These factors were only modestly correlated, suggesting that self-report and online confidence should be treated as independent constructs. Results of an EFA supported the hypothesis that self-report measures would be more closely related to personality and online measures would be more closely related to ability. There were significant differences between young and older adults on both confidence measures; for the self-report measures scores were about one standard deviation higher in older adults but for the online confidence measures a similar effect size was seen only for the measure of crystallized ability but not for the measures of fluid ability. An interesting and unexpected finding was that the self-report confidence measures appear to define something very similar to what is called Emotional Stability, a personality trait considered the opposite to Neuroticism. In this sample older adults scored substantially lower on Neuroticism than young adults (*d* = 1.32) and this effect size is similar to the differences seen on the measures of self-report confidence. Our results are consistent with the cross-sectional literature on Neuroticism (see McCrae et al., 2000) and consonant with longitudinal studies which show positive emotional experiences increase with age (Carstensen et al., 2011). Plausibly, what we have shown is that self-report confidence and self-reported emotional stability belong to the same broad domain; those who score low on Neuroticism are likely to express confidence about their lives in general. The online confidence measures showed that older adults were overconfident in both crystallized and fluid intelligence tasks; however, they were significantly more overconfident on one of the fluid tasks (APM) than the other two measures. Each individual hypothesis as well as the key aim will now be addressed in turn and discussed in further detail.

The main aim concerned the dimensionality of the five self-confidence measures (two self-report and three online). The results of a parallel roots analysis suggested a two factor solution; the two self-report confidence measures loaded on the first factor and the three online confidence measures loaded on the second factor. The entire sample was used in this factor analysis, controlling for age by regressing the variables which correlated with age (PEI, TROSCI, and WM) on age. This outcome has never been shown before and indicates that these measures cannot be considered to capture the same elements of self-confidence and should not be used interchangeably. An implication of this is that while it has been shown that online confidence is the best non-cognitive predictor of academic achievement, it is now clear that there is no substantial evidence to suggest that self-report measures can be used for this purpose. This should be considered further, however, because one study using the PEI (Cheng and Furnham, 2002) found that the academic confidence subscale but not actual academic performance, was predictive of happiness. The authors suggested that these self-report confidence measures may be useful in improving school performance and psychological wellbeing.

Hypothesis 1 was that both self-report and online measures of self-confidence are only modestly correlated with self-efficacy and personality, and self-confidence is an independent construct. This hypothesis was supported to the extent that both self-report and online confidence measures were on the whole positively correlated with self-efficacy and personality, with the exception of the personality trait Neuroticism, which had mostly negative relationships with both self-report and online measures. It cannot be said definitively that the self-report confidence measures define an independent construct, however; the correlations between Neuroticism and both the PEI and the TROSCI were very strong, indicating self-report confidence may actually be measuring something like Emotional Stability, a personality trait. One interpretation of the strong relationship between the TROSCI and Emotional Stability is that both measures are trying to capture stability of a construct (confidence and emotion respectively). However, the correlations between the PEI and Emotional Stability cannot be explained this way and, moreover, have been shown before in the development of the PEI scale; Schohn and Shrauger (1995) found a correlation of −0.60 between the general subscale of the PEI and Neuroticism. This is an important finding, which strongly supports the idea of a personality-related confidence derived from self-report measures of confidence. Specifically, the finding, replicated here, of a strong relationship between the PEI and Emotional Stability, and the additional parallel finding with the TROSCI, requires further investigation. It may be the case that when measuring self-report self-confidence, the construct being captured is largely Emotional Stability. The relationships of self-efficacy and personality with confidence were much stronger overall with the self-report than with the online measures. There were also some interesting differences between the young and older adults in terms of how the online confidence measures related to self-efficacy and personality. Correlations between online measures of confidence and the GSES and OCEANIC were consistently higher in the older adult sample. While in young adults the online measures had correlations close to zero with self-efficacy and most of the personality traits, correlations were in the small-to-moderate range for older adults; notably, a consistent pattern of relationships with the personality traits Extraversion, Agreeableness and Openness. It is unclear as to why this difference has emerged; however, it could be a possible effect of self-selection into the study by older adults, who, as willing volunteers, are likely to score higher than the general population on the personality traits Extraversion, Agreeableness and Openness.

Part (i) of Hypothesis 2 was that self-report measures of confidence are more strongly related to personality than are online measures. Part (ii) of Hypothesis 2 was that online measures of confidence are more strongly related to ability than are self-report measures of confidence.

An EFA was conducted including the five confidence measures (2 self-report and 3 online), the five personality traits, and the three ability measures; a three-factor model gave the best fit. Factor 1 was defined by the two self-report confidence measures (PEI and TROSCI), and three of the personality traits, Extraversion, Agreeableness and Neuroticism. Factor 2 was defined by the personality traits Openness, Conscientiousness, Extraversion and Agreeableness. Factor 3 was defined by the ability scores and online confidence measures. These findings provide clarification of the proposal that self-confidence sits on the no-man's land between ability and personality (Stankov, 1999). However, it is not, as was originally suggested, that a single construct sits between these two domains; rather, there are two separate constructs of confidence, one that sits closer to personality and one that sits closer to ability. The literature on Emotional Intelligence (EI) shows a similar phenomenon (see Matthews et al., 2009). It seems that questionnaire measures of EI have significant overlap with personality whereas the best-known ability based test of EI, the MSCEIT, has moderate correlations with general intelligence. The similarity between questionnaire versus ability based tests in both Confidence and EI measures suggest a pattern that merits further inquiry.

Hypothesis 3 was that there is a “general” confidence trait for online measures of self-confidence across tasks of both fluid and crystallized intelligence. A CFA modeling the three ability measures (WM, APM, & CAB-I) and their three respective confidence measures, found good fit for a two-factor model, where confidence measures loaded on the first factor and ability measures loaded on the second. The model was run first with the whole sample, controlling for age, and then with just the young adult sample. The model fit was acceptable in the whole sample and excellent in the young adult sample. These models also fit better than either a model that specifies all variables loading on a single factor, or a model with two factors representing fluid versus crystallized measures with both the ability score(s) and the confidence measure(s) loading on their respective factors. These analyses support Hypothesis 3, consistent with previous findings (see Kleitman and Stankov, 2007). The current study extends on previous literature because it was able to replicate the general confidence factor in a sample which included older adults. These findings are only preliminary and require replication using a larger older adult sample because the current sample size (*n* = 91) is marginal for factor analysis. Additionally, it would be useful to include more measures of a wider range of cognitive abilities, in order to clearly define a stable factor of online confidence.

There was also reason to suppose that self-report measures of confidence could also produce a confidence factor. At this point, it seems clear that any factor derived from self-report confidence measures would not be the same factor as produced by the online measures. Further, the suggestion of a general confidence factor defined by self-report measures was based on intercorrelations between the different confidence domains in a single self-report measure (PEI; Schohn and Shrauger, 1995); while this study considered the factor loadings of two different self-report measures. Nonetheless, the fact that the initial results of a parallel roots analysis suggested there were two separate factors, one defined by the self-report measures and one defined by the online measures indicates that the two self-report measures may define some kind of confidence factor. Given that there were only two self-report measures, this finding is only tentative; it would be useful to examine a larger number of self-report measures together for further clarification.

Part (i) of Hypothesis 4 was that the older adult sample has comparable or higher accuracy on measures of crystallized intelligence and better calibration than the young adult sample. This hypothesis was partially supported, accuracy on WM in the older adult sample was significantly higher (Cohen's *d* = 1.24, a large difference). Both groups were relatively well calibrated on the WM task but nonetheless, the difference between the groups was statistically significant (*p* = 0.002) and the effect size was moderate (*d* = 0.40). The older adults were not better calibrated as was anticipated.

Hypothesis 4 part (ii) was that the older adult sample has lower accuracy on measures of fluid intelligence, and an increase in calibration discrepancy. This hypothesis was supported for the APM, but surprisingly not for the CAB-I. For the APM, older adults had significantly poorer scores than the young adult sample (*d* = 0.73, a moderate-to-large effect). Similarly, the older adults were less well calibrated than the young adult sample (*d* = 0.85, a large effect). The CAB-I on the other hand had a pattern of results more consistent with an age comparison on a task of crystallized ability. There was no statistically significant difference between the groups on average percent correct (*d* = 0.13). Similarly, while it was anticipated that older adults would be more poorly calibrated, there was no significant difference between groups on calibration score (*d* = 0.06) and both groups were well calibrated.

Looking at both parts of Hypothesis 4 together (and disregarding the results of the CAB-I for this evaluation) suggests that older adults are more overconfident in judging their accuracy on ability tasks. To some extent, online self-confidence in older adults is related to the task it is attached to. The decline in fluid ability in older age is reflected in a pronounced overconfidence bias which is not seen so strongly in the crystallized ability task. This implies that while ability on fluid tasks has declined, there does not appear to be a metacognitive awareness of this. One interpretation of this is that online confidence remains stable across the lifespan regardless of decline in domain specific abilities; that is, where there is greater decline (such as in fluid ability) there will be greater overconfidence. This would explain why there is a substantial difference in overconfidence between fluid and crystallized ability tasks in older adults.

Further research into how the CAB-I behaves across age groups is necessary. This measure was chosen to measure fluid intelligence but the findings presented here are inconsistent with the literature on how fluid intelligence behaves across the lifespan. Given that the other measure of fluid intelligence used here (APM) has behaved as expected, there does not appear to be a problem with this sample. If this measure was a pure fluid ability measure there should have been significant group differences in favor of young adults on the percentage correct score. This finding may be a reflection on the fact that the CAB-I is a task that involves identifying patterns in sets of letters rather than of numbers, symbols, or visuo-spatial stimuli. It is plausible that the task may engage some degree of crystallized ability, explaining how it is that older adults did as well as young adults. A previous study (Kleitman and Stankov, 2007) used a measure of verbal reasoning, which was described as being dimensionally complex because it loaded on both fluid and crystallized intelligence factors and it is possible the CAB-I may function in a similar manner. Johnson and Bouchard (2005) in their development of a new model of intelligence used a battery of 42 tests which included the CAB-I. Results of a factor analysis showed the CAB-I loaded on both fluid and crystallized components of their VPR model of intelligence. These findings together suggest the CAB-I measure to be factorially complex and this seems to become more apparent when used with an older adult sample.

Based upon the findings presented above, the following can be concluded. Self-report and online confidence measures are both conceptually and empirically distinct, with self-report confidence measures sitting closer to personality traits (specifically Emotional Stability) and online confidence measures sitting closer to ability traits. There seems to be a stable confidence factor as defined by online confidence measures, this finding is consistent with previous literature; however, the current study was able to extend on this using an older adult sample. Young and older adults differ on confidence scores, especially the online measures yoked to crystallized and fluid ability tasks. In support of Crawford and Stankov (1996), older adults were overconfident on both tasks of crystallized (WM) and fluid intelligence (APM), however there was a substantially higher overconfidence bias in in the task of fluid intelligence than in the task of crystallized intelligence, which still sat within the ±10 parameters which define good calibration.

### Limitations

An underlying limitation of the current study was the use of Level 1 Psychology students. While this is a common occurrence in Psychological research, it is particularly problematic in the study of cognitive abilities measures. It is well known that for student populations there is a restriction of range in ability because students are selected on academic merit. In the current study this suggests a higher level of achievement on the ability tasks than would be expected in a general population sample. Additionally, the relationship between ability and confidence scores may also be impacted by this; however, the nature of this relationship has not been considered outside of a University student sample, so it is impossible to know how these variables function within the general population. The overall outcome being that any effects that depend on a full range of abilities may be under- or overestimated in the current study.

The study design was not particularly well suited to the older adult sample; participants were required to take part online from their own personal computer. For some older adults this medium is unfamiliar and as a result the task took considerably longer than for the young adults. One of the ways this impacted the data was through higher drop-out rates in the older adult sample. This is especially seen in the CAB-I, which has a smaller total *n* due to it being the final task. This could have been addressed by choosing less taxing or shorter form batteries when possible to lessen the participant burden, which was particularly apparent in the older adult group. Furthermore, the final sample size for the older adult sample was too small for us to conduct separate EFAs on the young and older adults and consequently we also could not test invariance of factor structures across the two groups. Of interest is the pattern of correlations between the confidence measures and abilities scores and between the confidence measures and the personality measures. For self-report confidence, 10-of-12 correlations with abilities scores were statistically indistinguishable, with only the correlations between TROSCI and CAB-I and TROSCI and APM differing between the groups. For the online confidence measures there were differences between groups in the correlations involving only APM (see Supplementary Table 3); these differed between groups for the correlations with APM and CAB-I scores. We also noted that while the highest correlation of the personality measures with self-report confidence was for Neuroticism, this relationship was stronger in young adults. Moreover, the correlation of Agreeableness with self-report confidence was near-zero in young adults and positive in older adults. We speculate that the factor structure reported in Table 5 is likely to be stable across groups; Factor 3 particularly is likely to be robust. The differing patterns of correlations with personality measures may imply, however, that Factor 1 might not be invariant across the two groups. The results in Table 5 should therefore be viewed as preliminary and a larger study is needed to settle these issues.

The cross-sectional design which was used to examine the way in which self-confidence functions across the lifespan is problematic in a number of ways. A longitudinal design would be desirable to truly see how the online confidence construct behaves across time. Comparing two separate age cohorts on this construct may be misleading in a similar way to a cross-sectional comparison of IQ; while the raw scores appear to decline when comparing age groups, the IQ score at an individual level remains relatively stable across the lifespan.

### Future research

The age comparison made in the current study provided some insight into how confidence behaves across the lifespan; however, replication with larger samples and a wider range of ability measures is necessary for further clarification of current findings. For both self-report and online measures of confidence young samples have been sourced from student populations, which as mentioned above can be problematic, especially for the online measures. It is necessary to consider how these measures behave in a general population sample. For the self-report measures of confidence, there is no previous literature using middle aged or older adult samples. Future research may consider using the currently developed self-report measures in wider samples, or the development of new self-report measures, using more representative samples.

### Conclusions

The findings presented here have implications for research in the area of self-confidence. It is clear that self-report and online measures cannot be used interchangeably; however, both measures may have important implications for improving academic achievement. It seems possible that the online confidence construct is stable across the lifespan, and the overconfidence bias seen especially in tasks of fluid ability in older adults is a reflection of a decrease in fluid ability across the lifespan.

## Author contributions

KB conducted the research as part of her Honors degree; she designed the study, collected and analyzed the data, and wrote the paper based on her Honors thesis. LW was the research supervisor and advised on all aspects of the study including the writing and data analysis and approved the final version of the paper. NB advised on the design of the study, the data handling and analysis, approved the final version of the paper, and is responsible for the analysis and results section of the final paper.

### Conflict of interest statement

The authors declare that the research was conducted in the absence of any commercial or financial relationships that could be construed as a potential conflict of interest.

## References

[B1] Asparouhov T. Muthén B. (2009). Exploratory structural equation modeling. Struc. Equ. Model. A Multidisc. J. 16, 397–438. 10.1080/1070551090300820419667138

[B2] Beattie S. Hardy L. Savage J. Woodman T. Callow N. (2011). Development and validation of a trait measure of robustness of self-confidence. Psychol. Sport Exerc. 12, 184–191. 10.1016/j.psychsport.2010.09.008

[B3] Betz N. E. Borgen F. H. (2010). The CAPA integrative online system for college major exploration. J. Career Assess. 18, 17–327. 10.1177/1069072710374492

[B4] Bors D. Stokes T. (1998). Raven's advanced progressive matrices: norms for first-year university students and the development of a short form. Educ. Psychol. Measurement 58, 382–399. 10.1177/0013164498058003002

[B5] Burns N. R. Bastian V. Nettelbeck T. (2007). Emotional intelligence: more than personality and cognitive ability? in Emotional Intelligence: Knowns and Unknowns, eds Matthews G. Zeidner M. Roberts R. D. (Oxford: Oxford University Press), 167–196.

[B6] Carstensen L. L. Turan B. Scheibe S. Ram N. Ersner-Hershfield H. Samanez-Larkin G. R.. (2011). Emotional experience improves with age: evidence based on over 10 years of experience sampling. Psychol. Aging 26, 21–33. 10.1037/a0021285 20973600 PMC3332527

[B7] Cattell R. B. (1966). The scree test for the number of factors. Multivariate Behav. Res. 1, 245–276. 10.1207/s15327906mbr0102_10 26828106

[B8] Cheng H. Furnham A. (2002). Personality, peer relations, and self-confidence as predictors of happiness and loneliness. J. Adolesc. 25, 327–339. 10.1006/jado.2002.0475 12128043

[B9] Costa P. T. McCrae R. R. (1985). The NEO Personality Inventory: Manual, Form S and Form R. Odessa, FL: Psychological Assessment Resources.

[B10] Crawford J. Stankov L. (1996). Age differences in the realism of confidence judgements: a calibration study using tests of fluid and crystallised intelligence. Learn. Individ. Differ. 8, 83–103. 10.1016/S1041-6080(96)90027-8

[B11] Hakstian A. R. Cattell R. B. (1975). The Comprehensive Ability Battery. Champaign, IL: Institute for Personality and Ability Testing.

[B12] Horn J. L. (1965). A rationale and test for the number of factors in factor analysis. Psychometrika 30, 179–185. 10.1007/BF02289447 14306381

[B13] Horn J. Noll J. (1994). A system for understanding cognitive capabilities: a theory and the evidence on which it is based, in Current Topics in Human Intelligence, Vol. 4, Theories of Intelligence, ed Detterman D. K. (Norwood, NJ: Ablex), 151–203.

[B14] Johnson W. Bouchard T. J. (2005). The structure of human intelligence: it is verbal, perceptual, and image rotation (VPR), not fluid and crystallized. Intelligence 33, 393–416. 10.1016/j.intell.2004.12.002

[B15] Jonsson A. C. Allwood C. M. (2003). Stability and variability in the realism of confidence judgments over time, content domain, and gender. Pers. Individ. Dif. 34, 559574 10.1016/S0191-8869(02)00028-4

[B16] Kavé G. Halamish V. (2015). Doubly blessed: older adults know more vocabulary and know better what they know. Psychol. Aging 30, 68–73. 10.1037/a0038669 25602490

[B17] Kleitman S. Costa D. (2014). The role of a novel formative assessment tool (Stats-mIQ) and individual differences in real-life academic performance. Learn. Individ. Differ., 29, 150–161. 10.1016/j.lindif.2012.12.001

[B18] Kleitman S. Moscrop T. (2010). Self-confidence and academic achievements in primary school children: their relationships and links to parental bonds, intelligence, age, and gender, in Trends and Prospects in Metacognition Research, eds Efklides A. Misailidi P. (New York, NY: Springer), 293–326.

[B19] Kleitman S. Stankov L. (2007). Self-confidence and metacognitive processes. Learn. Individ. Differ. 17, 161–173. 10.1016/j.lindif.2007.03.004

[B20] Kline R. (2011). Principles and Practice of Structural Equation Modeling, 3rd Edn. New York, NY: Guilford Press.

[B21] Matthews G. Deary I. J. Whiteman M. C. (2009). Personality Traits, 3rd Edn. Cambridge: Cambridge University Press.

[B22] McCrae R. R. Costa P. T. Jr. Lima M. P. Simoes A. Ostendorf F. Angleitner A.. (1999). Age differences in personality across the adult lifespan: parallels in five cultures. Dev. Psychol. 35, 466–477. 10.1037/0012-1649.35.2.466 10082017

[B23] McCrae R. R. Costa P. T. Jr. Ostendorf F. Angleitner A. Hrebícková M. Avia M. D.. (2000). Nature over nurture: temperament, personality, and life span development. J. Pers. Soc. Psychol. 78, 173–186. 10.1037/0022-3514.78.1.173 10653513

[B24] Morony S. Kleitman S. Lee Y. P. Stankov L. (2013). Predicting achievement: confidence vs self-efficacy, anxiety, and self-concept in Confucian and European countries. Int. J. Educ. Res. 58, 79–96. 10.1016/j.ijer.2012.11.002

[B25] Muthén B. Muthén L. (2014). Mplus Version 7: User's Guide. Los Angeles, CA: Muthén & Muthén.

[B26] Navarro D. J. (2015). Learning Statistics with R: A Tutorial for Psychology Students and Other Beginners. (Version 0.5) Adelaide, SA: University of Adelaide.

[B27] Pulford B. D. Sohal H. (2006). The influence of personality on HE students' confidence in their academic abilities. Pers. Individ. Dif. 41, 1409–1419. 10.1016/j.paid.2006.05.010

[B28] Raven J. Raven J. C. Court J. H. (1998). Manual for Raven's Progressive Matrices and Vocabulary Scales. Section 1: General Overview. San Antonio, TX: Harcourt Assessment.

[B29] R Core Team (2015). R: A Language and Environment for Statistical Computing. Vienna: R Foundation for Statistical Computing.

[B30] Sander P. Sanders L. (2003). Measuring confidence in a academic study a summary report. Elect. J. Res. Educ. Psychol. Psychoped. 1, 1–17.

[B31] Schohn M. Shrauger S. (1995). Self-Confidence in college students: conceptualization, measurement, and behavioral implications. Assessment 2, 255–278. 10.1177/1073191195002003006

[B32] Schulze R. Roberts R. (2006). Assessing the big five: development and validation of the openness conscientiousness extraversion agreeableness neuroticism index condensed (OCEANIC). Zeitschr. Psychol. 214, 133–149. 10.1026/0044-3409.214.3.133

[B33] Schwarzer R. Jerusalem M. (1995). Generalized Self-Efficacy scale, in Measures in Health Psychology: A User's Portfolio. Causal and Control Beliefs, eds Weinman J. Wright S. Johnston M. (Windsor: NFER-NELSON), 35–37.

[B34] Srivastava S. John O. P. Gosling S. D. Potter J. (2003). Development of personality in early and middle adulthood: set like plaster or persistent change? J. Pers. Soc. Psychol. 84, 1041–1053. 10.1037/0022-3514.84.5.1041 12757147

[B35] Stankov L. (1999). Mining on the “no man's land” between intelligence and personality, in Learning and Individual Differences: Process, Trait, and Content Determinants, eds Ackerman P. L. Kyllonen P. C. Roberts R. D. (Washington, DC: American Psychological Association), 315–337.

[B36] Stankov L. Crawford J. (1996a). Confidence judgments in studies of individual differences. Pers. Individ. Dif. 21, 971–986. 10.1016/S0191-8869(96)00130-4

[B37] Stankov L. Crawford J. (1996b). Confidence judgments in studies of individual differences support the ‘confidence/frequency effect’, in At Once Scientific and Philosophic: A Festschrift in Honour of JP Sutcliffe, eds Latimer C. Michell J. (Sydney, NSW: University of Sydney Press), 215–239.

[B38] Stankov L. Kleitman S. Jackson S. (2015). Measures of the trait of confidence, in Measures of Personality and Social Psychological Constructs, Boyle G. J. Saklofske D. H. Matthews G. (London: Academic Press), 158–189.

[B39] Stankov L. Lee J. Luo W. Hogan D. (2012). Confidence: a better predictor of academic achievement than self-efficacy, self-concept and anxiety? Learn. Individ. Differ. 22, 747–758. 10.1016/j.lindif.2012.05.013

[B40] Stankov L. Lee Y. P. (2008). Confidence and cognitive test performance. J. Educ. Psychol. 100, 961–976. 10.1037/a0012546

[B41] Stankov L. Morony S. Lee Y. P. (2013). Confidence: the best non-cognitive predictor of academic achievement? Educational Psychology 34, 9–28. 10.1080/01443410.2013.814194

