# Supplementary material for: Confidence—More a Personality or Ability Trait? It Depends on How It Is Measured: A Comparison of Young and Older Adults
Source: Front Psychol. 2016 Apr 18;7:518. doi: 10.3389/fpsyg.2016.00518 (PMC4834661; doi:10.3389/fpsyg.2016.00518)
Supplement: Supplementary file 1 [file Table1.PDF]

Supplementary Material

Confidence – More a Personality or Ability Trait? It Depends on How it is Measured: A Comparison of Young and Older Adults

Karina M. Burns[1], Nicholas R. Burns[1]*, Lynn Ward[1]

[1]School of Psychology, University of Adelaide, Adelaide, South Australia, Australia

*** Correspondence:** Nick Burns: nicholas.burns@adelaide.edu.au

Supplementary Table 1: Correlation matrix of all variables for the whole sample

1. GSES	1.	2.	3.	4.	5.	6.	7.	8.	9.	10.	11.	12.	13.	14.	15.	16.	17.
2. PEI	.50																
3. TROSCI	.39	.69															
4. O	.23	.05	.04														
5. C	.45	.28	.20	.22													
6. E	.47	.30	.21	.18	.26												
7. A	.32	.04	.01	.30	.38	.47											
8. N	-.36	-.70	-.72	.05	-.22	-.16	.01										
9. WM Corr	.14	.21	.28	.21	.23	.00	.08	-.26									
10. WM Conf.	.20	.34	.42	.19	.23	.04	.00	-.44	.81								
11. WM Calib	.09	.22	.24	-.04	.00	.06	-.14	-.29	-.30	.31							
12. APM Corr	.06	-.04	-.05	.09	-.02	-.05	.00	.13	-.01	-.12	-.17						
13. APM Conf.	.20	.17	.21	.11	.07	.08	.04	-.17	.14	.29	.24	.55					
14. APM Calib	.11	.21	.25	-.01	.09	.13	.04	-.30	.14	.4	.42	-.66	.26				
15. CAB Corr	.03	-.02	.00	.01	.05	-.17	.03	-.04	.31	.21	-.16	.46	.34	-.24			
16. CAB Conf.	.12	.10	.08	.02	.11	-.04	.09	-.16	.25	.33	.13	.36	.53	.05	.77		
17. CAB Calib	.12	.17	.10	.00	.07	.20	.08	-.16	-.13	.13	.43	-.21	.21	.44	-.48	.19	
Age	.01	.38	.43	.06	.13	-.13	-.16	-.53	.50	.62	.19	-.35	-.01	.40	.05	.08	.02

See Table 2 for full titles of previously mentioned abbreviated scales

Note: WM is Word Meanings, APM is Ravens Advanced Progressive Matrices, % Corr is Percentage correct, Conf Online is Confidence rating, Calib is Calibration.
