# Supplementary material for: Confidence—More a Personality or Ability Trait? It Depends on How It Is Measured: A Comparison of Young and Older Adults
Source: Front Psychol. 2016 Apr 18;7:518. doi: 10.3389/fpsyg.2016.00518 (PMC4834661; doi:10.3389/fpsyg.2016.00518)
Supplement: Supplementary file 2 [file Table2.PDF]

Supplementary Material

Confidence – More a Personality or Ability Trait? It Depends on How it is Measured: A Comparison of Young and Older Adults

Karina M. Burns[1], Nicholas R. Burns[1]*, Lynn Ward[1]

[1]School of Psychology, University of Adelaide, Adelaide, South Australia, Australia

*** Correspondence:** Nick Burns: nicholas.burns@adelaide.edu.au

Supplementary Table 2: Correlation matrix comparing young and older adult samples

	1.	2.	3.	4.	5.	6.	7.	8.	9.	10.	11.	12.	13.	14.	15.	16.	17.
1. GSES		.58	.46	.11	.47	.42	.20	-.52	.14	.23	.09	.04	.09	.04	.00	.12	.16
2. PEI	.45		.67	-.03	.26	.33	.03	-.69	.02	.20	.22	.13	.17	-.01	-.02	.15	.23
3. TROSCI	.35	.52		-.04	.15	.19	.00	-.72	.12	.31	.22	.2	.23	-.03	.07	.19	.14
4. O	.42	.12	.10		.16	.04	.19	.13	.23	.12	-.16	.14	.08	-.10	-.01	-.04	-.04
5. C	.41	.20	.17	.31		.27	.37	-.19	.17	.13	-.07	.00	-.03	-.03	.01	.02	.01
6. E	.56	.50	.49	.43	.31		.41	-.20	.01	.05	.04	-.17	-.09	.14	-.16	-.02	.22
7. A	.56	.28	.26	.55	.50	.54		-.03	.21	.09	-.18	-.09	-.04	.07	.04	.08	.04
8. N	-.17	-.49	-.47	.01	-.12	-.46	-.26		-.04	-.22	-.23	-.16	-.28	-.08	-.11	-.27	-.18
9. WM % Corr	.17	.00	-.02	.18	.20	.19	.16	.11		.71	-.48	.17	.10	-.12	.33	.21	-.23
10. WM Conf.	.24	.02	.05	.30	.27	.34	.21	-.03	.79		.28	.12	.32	.18	.28	.39	.09
11. WM Calib	.07	.02	.10	.16	.09	.19	.05	-.22	-.43	.21		-.08	.26	.38	-.11	.20	.44
12. APM % Corr	.12	.02	-.09	.09	.11	.05	-.01	.23	.23	.11	-.21		.68	-.63	.54	.43	-.27
13. APM Conf.	.37	.23	.23	.15	.25	.35	.19	-.02	.26	.44	.23	.45		.15	.44	.63	.14
14. APM Calib	.21	.18	.30	.04	.12	.27	.18	-.26	.00	.28	.42	-.60	.44		-.26	.08	.51
15. CAB % Corr	.08	-.11	-.25	.03	.13	-.17	.04	.37	.29	.10	-.35	.43	.13	-.33		.76	-.54
16. CAB Conf.	.10	-.09	-.25	.09	.26	-.07	.16	.27	.30	.26	-.09	.38	.35	-.07	.80		.14
17. CAB Calib	.04	.03	.00	.09	.20	.16	.18	-.15	.01	.27	.41	-.09	.35	.41	-.33	.31	

Note: top right corner: young adults, bottom left corner: older adults.
