# Supplementary material for: Confidence—More a Personality or Ability Trait? It Depends on How It Is Measured: A Comparison of Young and Older Adults
Source: Front Psychol. 2016 Apr 18;7:518. doi: 10.3389/fpsyg.2016.00518 (PMC4834661; doi:10.3389/fpsyg.2016.00518)
Supplement: Supplementary file 3 [file Table3.PDF]

Supplementary Material

Confidence – More a Personality or Ability Trait? It Depends on How it is Measured: A Comparison of Young and Older Adults

Karina M. Burns[1], Nicholas R. Burns[1]*, Lynn Ward[1]

[1]School of Psychology, University of Adelaide, Adelaide, South Australia, Australia

*** Correspondence:** Nick Burns: nicholas.burns@adelaide.edu.au

Supplementary Table 3: Correlations and 95% confidence intervals for percentage accuracy scores for WM, APM and CAB-I with both self-report and online measures of self-confidence for young (n = 153) and older adults (n = 91).

	Self-report Self-confidence						Online Self-confidence			
	PEI		TROSCI		Word Meanings		APM		CAB-I	
Young Adults										
WM % correct	.02	[-.14, .18]	.12	[-.04, .28]	.71	[.62, .78]	.10	[-.06, .26]	.21	[.05, .36]
APM % correct	.13	[-.02, .29]	.20	[.05, .35]	.12	[-.04, .27]	.68	[.58, .75]	.43	[.29, .55]
CAB-I % correct	-.02	[-.18, .14]	.07	[-.10, .23]	.28	[.12, .42]	.44	[.30, .56]	.76	[.68, .82]
Older Adults										
WM % correct	.00	[-.20, .21]	-.02	[-.23, .19]	.79	[.69, .85]	.26	[.06, .44]	.30	[.08, .49]
APM % correct	.02	[-.18, .23]	-.09	[-.29, .12]	.11	[-.10, .31]	.45	[.27, .60]	.38	[.17, .56]
CAB-I % correct	-.11	[-.33, .12]	-.25	[-.45, -.03]	.10	[-.13, .31]	.13	[-.10, .34]	.80	[.70, .87]
